# The Short-Chain Fatty Acid Acetate in Body Weight Control and Insulin Sensitivity

**DOI:** 10.3390/nu11081943

**Published:** 2019-08-18

**Authors:** Manuel A. González Hernández, Emanuel E. Canfora, Johan W.E. Jocken, Ellen E. Blaak

**Affiliations:** Department of Human Biology, NUTRIM School for Nutrition and Translational Research in Metabolism, Maastricht University Medical Centre+, Universiteitssingel 50, P.O. Box 616, 6229 ER Maastricht, The Netherlands

**Keywords:** acetate, dietary fiber, microbiota, obesity, type 2 diabetes

## Abstract

The interplay of gut microbiota, host metabolism, and metabolic health has gained increased attention. Gut microbiota may play a regulatory role in gastrointestinal health, substrate metabolism, and peripheral tissues including adipose tissue, skeletal muscle, liver, and pancreas via its metabolites short-chain fatty acids (SCFA). Animal and human data demonstrated that, in particular, acetate beneficially affects host energy and substrate metabolism via secretion of the gut hormones like glucagon-like peptide-1 and peptide YY, which, thereby, affects appetite, via a reduction in whole-body lipolysis, systemic pro-inflammatory cytokine levels, and via an increase in energy expenditure and fat oxidation. Thus, potential therapies to increase gut microbial fermentation and acetate production have been under vigorous scientific scrutiny. In this review, the relevance of the colonically and systemically most abundant SCFA acetate and its effects on the previously mentioned tissues will be discussed in relation to body weight control and glucose homeostasis. We discuss in detail the differential effects of oral acetate administration (vinegar intake), colonic acetate infusions, acetogenic fiber, and acetogenic probiotic administrations as approaches to combat obesity and comorbidities. Notably, human data are scarce, which highlights the necessity for further human research to investigate acetate’s role in host physiology, metabolic, and cardiovascular health.

## 1. Introduction

Obesity has reached pandemic proportions worldwide, and its increased prevalence is associated with a plethora of metabolic disturbances [1]. The obese state is characterized by increased adipose tissue mass and disturbed function resulting in systemic lipid spillover and low-grade inflammation, which may contribute to the development of comorbidities such as type 2 diabetes mellitus (T2DM) and cardiovascular disease [2,3,4]. The crosstalk between various metabolic organs such as the gut, liver, adipose tissue, and skeletal muscle plays an important regulatory role in energy and substrate metabolism, which impacts metabolic health [5].

In recent decades, the role of the gut microbiota in host energy and substrate metabolism has been under extensive investigation [6,7,8]. This includes interventions that modify the gut microbiota composition and functionality with antibiotics [9,10], prebiotics [11,12], probiotics [13], and postbiotics [14]. Additionally, gut microbes are able to ferment indigestible foods, such as dietary fibers, which, thereby, yields short-chain fatty acids (SCFA) as end products that may confer beneficial metabolic effects [15,16,17]. In general, saccharolytic fermentation mostly occurs in the distal ileum and proximal colon. The most abundant SCFA are acetate, propionate, and butyrate with an approximate molar ratio of 60:20:20, respectively [18,19,20]. In mice, the cecum has been described as a major site of SCFA production [21]. After colonic absorption and transition to the systemic circulation, the molar ratio changes to approximately 91:5:4, respectively, which are numbers that are based on findings in sudden death victims [22].

Acetate may act by binding to the G-protein coupled receptors (GPR), GPR43 (FFAR2), and GPR41 (FFAR3), which are expressed at the mRNA and protein level in the human colon [23,24] but are also expressed in the small intestine, such as, in particular, the ileum [25]. Moreover, these receptors have been shown to be expressed at the mRNA level in various insulin sensitive tissues such as the adipose tissue [26], skeletal muscle, liver [27], and pancreatic beta cells [28,29], which illustrates their broad metabolic role. Intracellularly, however, acetate may be converted to acetyl-CoA and incorporated in the tricarboxylic acid (TCA) cycle in various peripheral tissues [30,31,32]. It may also impact metabolism through increments in oxidative capacity (e.g., liver and skeletal muscle) via effects on 5′AMP-activated protein kinase (AMPK) phosphorylation [33,34,35]. Acetate may also increase fatty acid synthesis through epigenetic mechanisms such as histone acetylation [36]. Additionally, acetate may activate other receptors important for blood pressure regulation including olfactory receptors 51E2 (Olfr51E2) and 78 (Olfr78) in renal tissue [37]. In this review, we focus on the metabolic effects of the most abundant (in the colon and systemic circulation) gut-derived metabolite, known as acetate, which may improve the obese insulin resistant state through various effects in peripheral tissues that collectively improve body weight control and insulin sensitivity.

We provide an overview of recent literature on dietary sources of acetate, gut-derived acetate production/absorption after fiber fermentation, and prebiotics and probiotics that may increase plasma acetate through colonic fermentation. We will discuss the available literature on the effect of acetate on body weight control (central effects of appetite regulation and satiety hormones and energy expenditure) as well as its role in insulin sensitivity in the context of the metabolic inter-organ cross-talk between skeletal muscle, liver, and adipose tissue metabolism [38,39,40,41]. Additionally, acetate effects on insulin secretion will be discussed. With respect to acetate effects on cardiovascular health, data is limited and the role of the gut microbiome on cardiovascular health has been reviewed elsewhere [42,43]. Lastly, we discuss potential therapeutic approaches to improve insulin sensitivity and metabolic health, including oral acetate administration (vinegar intake), acetogenic fiber, and probiotic supplementations.

## 2. Dietary Sources and Gut-Derived Acetate Production and Absorption

### 2.1. Acetate from Dietary Sources

According to the Codex General Standard for Food Additives [44], acetate is present in dietary components as an acidity regulator (pH control agent), preservative, or sequestrant. For instance, acetate-containing foods include dairy products, dried pastas, bread, liquid eggs, salt substitutes, coffee, coffee substitutes, processed meat, and smoked/frozen fish [44]. Other important sources are ethanol [45] and vinegar [46]. Commonly consumed vinegars contain between 4% and 8% of acetic acid, and vinegar ingestion has gained attention because of its acute effects in glucose and lipid metabolism, as extensively reviewed by Lim et al. [46].

Oral ingestion of vinegar rapidly increases circulating acetate as observed in healthy participants that increased serum acetate levels from 120 µmol/L during placebo conditions up to 350 µmol/L (after 15 min) and 200 µmol/L (after 30 min) after vinegar (100 mL containing 0.75 g acetic acid) and acetic acid capsules (containing 0.75 g of acetic acid) intake, respectively [47]. Acetic acid is a bioactive component with a dominant flavor in different types of vinegars including cider, malt, plum, sherry, tomato, and wine vinegar [48].

In addition, vinegars may contain other polyphenol residual components (e.g., gallic acid, catechin) such as in apple cider, grape, sherry, and Balsamic vinegar [48]. Therefore, it is important to consider the vinegar type since their composition of phenolic, flavonoid, and acetic acid content may differ [49]. In general, various dietary products such as preservatives, acidity regulators, food substitutes, ethanol, and vinegar may provide acetate orally. In particular, vinegar may provide rapid increments in plasma acetate levels due to its fast absorption in the upper digestive tract (See vinegar administrations in humans). However, future vinegar supplementations should specify detailed composition including acetic acid percentage and polyphenols content.

### 2.2. Microbial-Derived Acetate Production

Microbial-derived acetate production is yielded by the fermentation of indigestible foods especially foods of acetogenic fibers (e.g., galacto-ligosaccharides, inulin) [50]. In postprandial conditions, acetogenic fibers can be fermented and may elevate production of acetate in the proximal colon (see acetogenic fibers in human studies) [51]. When acetogenic fibers reach the colon, acetate is mainly generated by the microbial community via two metabolic pathways: acetogenesis and the carbon fixation pathway [52]. Acetogenesis is the production of acetate, mediated by homoacetogenic bacteria or acetogens (found in the digestive tract of humans and ruminants), which are capable to produce acetate from H_2_ and carbon dioxide (CO_2_) [53]. The carbon fixation pathway (also known as Wood-Ljungdahl pathway) produces acetate from CO_2_ as a precursor [52].

In addition, acetate may originate from microbial fermentation of residual peptides and fats [54,55,56]. For instance, high fat diet (HFD)-fed rats (60% fat) showed elevated colonic and whole-body acetate turnover together with a shift on the phylum level (increased *Firmicutes/Bacteroidetes* ratio) [57]. Additionally, microbiota transplant of HFD-fed to germ-free rats increased acetate turnover [57]. In a Western diet (low fiber intake), protein fermentation occurs mainly in the distal colon where saccharolytic substrates are depleted [58] and this produces other compounds of toxic nature such as ammonia, amines, phenols, and sulfides [59]. Branched chain and aromatic amino acids may be produced and further metabolized via cross-feeding mechanisms and alter gut integrity and impair insulin sensitivity [60]. In summary, gut-derived acetate production is determined by the balance between saccharolytic and proteolytic fermentation and is especially determined by the presence of acetogenic fibers. It is tightly regulated by the intricate interplay within the microbial community.

Although gut-derived acetate production is expected to be low during states of low presence of fibers such as fasting, some studies suggest a possible contribution of fasting-induced alterations in the gut microbiota to fasting acetate concentrations [61]. This was accompanied by an increase in the *Firmicutes/Bacteroidetes* ratio and cross-feeding mechanisms as shown by an upregulation of pyruvate fermentation pathways to acetate and lactate by *Lactobacillus reuteri* and other unclassified bacteria [61]. In support, human fasting and caloric restriction interventions have described an increase in microbial diversity and abundance of important acetate producers, such as *Akkermansia Muciniphila (A. muciniphila)* and *Bifidobacteria* [62,63].

### 2.3. Colonic and Systemic Acetate Concentrations

Acetate concentrations in the colon start with the highest levels in the caecum (69 mmol/L) and ascending colon (63 mmol/L), which was followed by a subsequent decrease in the transverse colon (57.9 mmol/L), descending colon (43.5 mmol/L), and sigmoid colon (50.1 mmol) (measured by kilograms of intestinal luminal contents) as observed in sudden death victims [22]. This progressive decline along the colon suggests that major acetate production and absorption occurs in the proximal colon. In general, microbially produced acetate in the proximal colon may follow a colonic-hepatic-periphery distribution starting with colonic levels in the mmol/L range, which was followed by a significant drop, around 10-fold in the liver, and reaching the periphery in the µmol/L range (See Table 1) [22].

A recent study that sampled acetate simultaneously from different colonic sites (proximal, distal), inferior/superior mesenteric veins (IMV/SMV), portal/hepatic vein, and radial artery in patients undergoing surgery found acetate release was the highest in the IMV (102.7 ± 27.2 µmol/L) and lowest in the radial artery (21.8 ± 7.6 µmol/L). They reported a correlation of arterial acetate concentrations with those in the IMV (*r*^2^ = 0.65, *p* < 0.01), not with SMV, and much lower concentrations in hepatic and portal veins (23.6 ± 4.8 µmol/L and 41.4 ± 7.8 µmol/L, respectively). Collectively, this suggested a greater release from the distal colon [64]. This highlighted the distal colon as a potential location to promote acetogenic fiber fermentation and/or highlighted it as the location to test the effectiveness of a fiber supplementation in future studies. A rodent study identified increments in circulating acetate concentrations after intraperitoneal infusion and oral gavage, but not after acetate administration in drinking water [66]. Nevertheless, some studies that provided acetate in drinking water have reported beneficial effects [67,68,69]. However, the study [66] suggests that acetate effects/kinetics and clearance may depend on the administration site of exogenous acetate. However, it should be mentioned that this study did not measure acetate with the gold standard technique (electron ionization gas chromatography mass spectroscopy). Instead, it used a commercially available colorimetric kit that allowed the measurement of multiple time points (each measurement required only ~1 µL of plasma) [66]. In summary, acetate production and colonic acetate release may vary along the colon. Thus, the site of fermentation and acetate production may be an important determinant of circulating concentrations.

### 2.4. Prebiotics and Bacterial Acetate Producers

#### 2.4.1. Prebiotic In Vitro Studies

First, using an in vitro system mimicking colonic digestion [70], researchers reported that fermentation of starch-entrapped microspheres, fructo-oligosaccharides (FOS), and Psyllium led to substantial in vitro acetate production at 24 h and 48 h (140 and 211, 187 and 231, 178 and 219 µmol/50 mg carbohydrate, respectively). However, corn bran arabinoxylans in the same system showed a higher acetate production after 24 and 48 h (222, 284 µmol/50 mg carbohydrate, respectively) [71]. Furthermore, using the proximal large intestine TNO in vitro model (TIM-2) (developed by TNO, Zeist, The Netherlands), the addition of the acetogenic fiber galacto-oligosaccharides for 72 h increased total SCFA (231 vs. 144 control mmol/L, respectively) and acetate production (±49%) to approximately 500 µmol/50 mg of carbohydrates [72]. Another in vitro study, comparing FOS vs. inulin mixtures with various degrees of polymerization (DP) reported that a mixture of Inulin (90–94% DP > 10 and 6–10% DP = 1–2) showed the greatest acetate levels at 24 h (25.1 mmol/L) and a mixture of Oligofructose (>93.2% DP < 10 and <6.8% DP = 1) at 4 h increased the levels (39.7 mmol/L). However, this not reach a significance [73]. Similarly, a comparison of Inulin (DP 3–60) vs. Oligofructose (DP 2–20) with various DP using the Simulator of Human Intestinal Microbial Ecosystem (SHIME) in vitro model, reported that inulin with longer chain lengths (e.g., Chicory) showed a slower breakdown, more beneficial changes in microbial community (both proximal and distal), higher acetate production, and higher bifidogenic effect in the distal colon than oligofructose [74]. In addition, the glycosidic bond configuration of dietary fibers may affect acetate production [75]. For instance, different glycosidic bonds in the β orientation led to differences in acetate production (diglucose β 1-6 vs. diglucose β 1-4, 3.7 ± 0.3 vs. 1.9 ± 0.2 mmol/g carbohydrate/day, *p* = 0.001), but it did not differ between fibers with various glycosidic bond configurations (α and β) [76]. With respect to timing, a recent in vitro study concluded that the fermentation was delayed more when a mixture was used (arabinoxylan, chondroitin sulfate, galatomannan, polygalactunoric acid, and xyloglucan) rather than when the individual fibers were provided. They suggested that mixtures of fibers may promote the late fermentation of soluble fibers in the distal colon and prevent deleterious effects of proteolytic activity [77]. Collectively, these studies have demonstrated that, by the use of in vitro fermentation models, the potential of prebiotic fibers as well as the importance of fiber characteristics (e.g., DP, glycosidic bond configuration, orientation, chain length) produce acetate. However, future studies are needed to investigate whether these results on delayed colonic acetate production can be replicated in humans and to what extent acetate is further absorbed and metabolized in humans.

#### 2.4.2. Bacterial Acetate Producers

Acetate production is widely distributed within the bacterial community, since many bacterial species are acetate producers [78]. However, some species seem to be major acetate producers. For instance, humanized gnotobiotic mice that were co-colonized with *Bacteroides Thetaiotaomicron/Methanobrevibacter smithii* reported higher serum acetate levels as compared to *Bacteroides Thetaiotaomicron* alone [79]. In overweight/obese individuals, a positive association between faecal *A. muciniphila* and serum acetate (*r*^2^ = 0.36, *p* = 0.01) at baseline was described. However, six-week caloric restriction resulted in decreased serum acetate levels and disappearance of the associations, which indicated sensitivity to diet changes [63]. Of importance, differences in the abundances of species along the colon may contribute to differences in acetate production between the proximal and distal colon [80,81]. According to the SHIME in vitro model, *A. muciniphila* colonization preferred high pH (6.6–6.9) and high concentration of mucin, which are more abundantly present in the distal colon compared to the proximal colon [82]. In support, murine studies have reported higher abundance of *A. muciniphila* in the colon as compared to the ileum [83,84]. Moreover, in vitro models that mimic the human colon reported the highest concentration of *A. muciniphila* in the transverse compartment, which was followed by the descending compartment and no detection in the ascending colon [85]. Further discussion on important acetate microbial producers is continued under therapeutic approaches (See probiotics, body weight control, and insulin sensitivity)**.**

## 3. Acetate in Body Weight Control

Acetate administration or microbially-derived acetate may have an impact on body weight control through effects on energy intake as well as energy expenditure. In a 12-week intervention in HFD-fed mice, Den Besten et al. [86] found that oral sodium acetate supplementation incorporated into the diet (at 5%, *weight*/*weight.* proportion) resulted in a suppression of HFD-induced weight gain (~30%) compared to control mice fed an HFD [86]. Similarly, Lu et al. [87] showed that 16-weeks of oral sodium acetate supplementation (5%, *wt.*/*wt.*) suppressed HFD-induced weight gain by 72% (*p* < 0.05) in comparison to control mice fed an HFD. In addition, a six-week intragastric administration of acetic acid (50, 250 mmol/L) to HFD-fed mice reduced weight gain (7% and 8%, respectively) and body fat accumulation in comparison to HFD alone [88]. In contrast, Perry et al. [57] described that both continuous intragastric acetate infusions (10 days, at a rate of 20 µmol kg^−1^ min^−1^) and an HFD (three days and four weeks) in rats led to similar increased systemic ghrelin, gastrin concentrations, and glucose stimulated insulin secretion (GSIS) that collectively promoted hyperphagia and energy retention that ultimately caused weight gain. This elegant rodent study provided valuable mechanistic insight in the acetate role in metabolic health even though conflicting with other important rodent studies [86,87,88,89]. The differences might be related to the species and phenotype of animals used (e.g., Sprague-Dawley rats versus C57BI/6J mice) or administration site (intragastric versus oral or colonic administration). This further highlights the need to investigate metabolic effects of an increased whole-body acetate turnover in humans. In contrast to the outcomes of Perry et al., a recent human study conducted by the same group failed to corroborate these findings, which reported a higher acetate turnover in lean (~30%) versus obese individuals and found no effect of increased circulating acetate levels (intravenous infusion) on ghrelin and GSIS in obese individuals [90]. These important data values showed that there are important differences across species and between modes of administrations.

In humans, long-term oral acetate supplementation or intravenous/gastric/colonic infusion studies with weight loss and energy expenditure as the primary outcome are limited (See acetate effects on energy expenditure). Cross-sectional/cohort analyses have shown inconsistent results with obesity and adiposity. For instance, one study showed that fasting acetate correlated negatively with visceral adipose tissue mass in obese women [91]. In contrast, in obese men and women, a positive association (r^2^ = 0.11, *p* = 0.004) of fasting plasma acetate (0–2 µmol/L range) with a degree of adiposity at baseline including total body fat, visceral fat, and subcutaneous fat (magnetic resonance imaging) was observed independent of age, sex, and ethnicity [92]. In addition, baseline fasting plasma acetate positively predicted changes in adiposity (delta body mass index (BMI) per year) after a 2.2 ± 1.7-year follow-up time independent of baseline BMI, age, sex, and ethnicity. Additionally, fasting plasma acetate levels correlated positively with de novo fasting hepatic lipogenesis (measured by hepatic palmitate incorporation) [92]. As mentioned above, long-term oral acetate supplementation studies in humans are lacking and data on the role of acetate in body weight management are mainly based on animal data and human cross-sectional data. The above discrepancies highlight that acetate effects on body weight control may depend on the mode of administration, metabolic phenotype, and species-specific differences in acetate metabolism. In the following paragraphs, we discuss the putative mechanisms of how acetate might influence body weight control via the central nervous system mechanisms and gut-derived hormones as well as via effects on energy expenditure.

### 3.1. Acetate and Central Effects on Appetite Regulation

Appetite regulation is coordinated by nutrients and microbial metabolites through the central nervous system circuitry and circulating hormones from peripheral tissues [93]. Acetate has been reported to cross the blood-brain barrier in both mice [89] and humans [94]. Additionally, acetate has been detected in the cerebrospinal fluid, which suggests a central homeostatic mechanistic role [95]. In support, Frost et al. [89] described an association of elevated colonic acetate and a direct role of acetate on appetite regulation in HFD-fed mice supplemented for eight weeks with acetogenic oligofructose-enriched inulin. Acetate accumulation in the hypothalamus was shown to affect appetite regulation through the glutamate-glutamine transcellular cycle, which resulted in increments in lactate and gamma aminobutyric acid (GABA) production, after both intraperitoneal acetate injections and after colonic fermentation of ^13^C-labelled carbohydrate [89]. The mice showed a peak in serum acetate levels (350 µmol/L) 10 min after an intraperitoneal acetate injection (500 mg/kg^−1^), which was associated with changes in the expression of neuropeptides (AMPK and acetyl CoA carboxylase (ACC) that regulate appetite suppression) [89]. Hypothalamic acetate administration showed inactivation of AMPK and activation of ACC (via decreased phosphorylation), which suggests that acute administration of acetate may increase hypothalamic ACC activity. ACC activation may increase malonyl-CoA, which may lead to a reduction in food intake through the expression of orexigenic and anorexigenic neuropeptides in the hypothalamus via two mechanisms: (i) via the interaction of malonyl-CoA with signaling proteins or (ii) via inhibition of carnitine/palmitoyl-CoA transferase (CPT) that prevents the entry of the long-chain fatty acids to the mitochondrion [96,97]. These data values are of an associational nature. Therefore, to assess a causal role of these pathways and to further study the role of acetate on appetite and satiety regulation animal models (e.g., MC4R/mice) might be used.

Acetate signaling in the brain may follow a hepatic-portal-vagal route. However, vagal activation and effects on body weight control may depend on the site of administration and use of different animal models [98]. For instance, Perry et al. [51] reported that both HFD and continuous intragastric infusions of acetate increased central vagal activation, including a vagal-induced release of ghrelin from the stomach that led to a metabolic syndrome phenotype in rats, as explained above. In mice, an intraperitoneal acetate injection (6 mmol/kg) significantly reduced food intake (0.5 and 1 h after administration) in a vagal-dependent manner since vagotomy attenuated the effect [99]. Although these studies may imply that vagal activation in response to acetate are contradictory, it is important to note that the site of administration and use of different animal models may explain the difference in vagal activation. In addition, energy intake may also regulate vagal activation by gut hormones (GLP-1, glucagon like peptide 1, and peptide YY, PYY), as vagotomized humans showed impaired effects of exogenous GLP-1 on food intake [100]. In summary, acetate may regulate appetite possibly through central hypothalamic mechanisms and satiety through acetate-induced or gut hormone-induced vagal activation. However, due to the lack of human evidence and the inconsistency in animal data, further research should elucidate the exact underlying mechanism.

### 3.2. Gut-Derived Satiety Hormones

As indicated above, gut-derived satiety hormones can be secreted from enteroendocrine cells located in the gut [101]. Enteroendocrine cells, in particular the L-cells, secrete GLP-1 and PYY hormones that seem to play an important role in gut health and in the connection of the gut-brain axis [102]. Notably, both acetate receptors GPR43 [23] and GPR41 [24] are expressed at mRNA and the protein level in enteroendocrine cells in the human colonic mucosa, which potentially indicates an acetate-mediated effect in their secretion [103]. In support, in vitro culturing of enteroendocrine cells (SCT-1 cell line) with acetate (3 and 30 mmol/L) for 24 h showed increased expression of proglucagon (GLP-1 precursor) in a dose-dependent manner [104]. In a mice study, inulin supplementation was protected from HFD-induced obesity, which was dependent on an increased PYY secretion in a GPR43 dependent manner [105]. In addition, dietary fiber supplementation in rats with resistant starch (RS) increased plasma levels of GLP-1 and PYY in the short term (24-h period) [104]. Similarly, rats supplemented with RS (30-day period) increased plasma levels of GLP-1 and PYY and increased caecal acetate [106]. Using inulin type fructans, Cani et al. [107] demonstrated that a three-week oral supplementation in rats increased GLP-1 and decreased ghrelin plasma levels, possibly via colonic SCFA production especially acetate. In addition, feeding of dogs with a mixture of high fermentable fibers for 14 days increased plasma GLP-1 concentration 15 min after an oral glucose load. However, systemic acetate was not measured [108]. Importantly, GPR41 has been reported in other enteroendocrine cell types (secretin and neurotensins types) in the duodenum and proximal colon of mice, respectively [25]. However, whether acetate can induce a GPR41-dependent secretion of these appetite-suppressing hormones needs further investigation.

In humans, production of gut-derived satiety hormones in response to prebiotic supplementation is scarce and inconclusive. For instance, Rozenbloom et al. [109] reported an increase in colonic acetate but no increase in serum levels of satiety hormones (GLP1, PYY) in overweight individuals after inulin ingestion (single dose 24 g) in comparison to glucose as the control. In contrast, oligofructose supplementation (21 g/day) for 12 weeks in overweight/obese individuals increased the area under the curve (AUC) of PYY, and decreased AUC of ghrelin secretion. This coincided with a reduction in self-reported caloric intake and was associated with a significant weight loss (1.03 ± 0.43 kg) as compared to maltodextrin [110]. In addition, oligofructose (35 g/day) in lean individuals, increased postprandial PYY concentrations [111]. Importantly, these human prebiotic interventions did not measure fecal/systemic acetate.

Another approach to investigate secretion of gut-derived hormones is through colonic acetate infusions. Infusions in humans in different sites may elicit differential effects on gut-derived satiety hormones. For example, rectal (60 mmol/L) sodium acetate administrations increased PYY and GLP-1 significantly as compared to intravenous infusions (acetate 20 mmol/L and saline) [112]. Similarly, distal (not proximal) colonic infusions of sodium acetate (180 mmol/L) increased PYY in overweight/obese men and only 180 mmol/L (not 100 mmol/L) elicited these effects [39]. Moreover, rectal/intravenous infusions showed the potential to induce gut-derived hormone secretion (GLP-1 and PYY) in a metabolically disturbed phenotype [113]. Together, these distal colonic infusions studies suggest the relevance of the site of administration or fermentation (distal versus proximal colon) to modulate gut hormone secretion. Of note, a higher density of PYY producing cells in the distal colon in rodent studies [114,115] may explain the differential effect on hormonal secretion dependent on the site of fermentation.

Acetate may also increase the secretion of leptin from the adipose tissue. For instance, in a rodent study, acetate (860 µmol/L) increased transcription of the leptin gene, and propionate (78 µmol/L) showed even stronger effects [116]. In bovine adipocytes, acetate (1 mmol/L) increased leptin expression by ~60%, which was inhibited by pertussis toxin and indicates GPR dependence [117]. Further studies have to show the relevance of this mechanism under in vivo conditions. In summary, animal and in vitro data studies have shown the potential of acetate to increase gut hormone secretion. However, dietary fiber supplementation in humans have shown inconsistent results. Future human trials may aim to target distal colonic fermentation since distal colonic acetate infusions have shown pronounced increments in gut satiety hormones.

### 3.3. Acetate Effects on Energy Expenditure

A few studies have investigated the direct effects of acetate on energy expenditure as a primary outcome. Acetate has been related to increments in energy expenditure through various mechanisms in peripheral tissues [41] (See Acetate inter-organ crosstalk and insulin sensitivity in peripheral tissues). In this section, we discuss the effects of acetate infusions in humans and also in the form of vinegar through oral administrations in the context of body weight control.

#### 3.3.1. Acetate Infusions in Humans and Energy Expenditure

In humans, a lipid lowering effect of acetate after rectal infusions [118] and intragastric [119] infusions in healthy subjects almost three decades ago paved the way for future human acetate infusions (See Table 2). Recently, acute colonic infusions showed increments in fasting fat oxidation and energy expenditure in humans [39,120]. First, distal colonic infusions in normoglycemic overweight/obese individuals increased fasting fat oxidation (1.78 ± 0.28 vs. −0.78 ± 0.89 g fat 2 h^−1^, *p* = 0.015). However, there were no effects on energy expenditure [39]. Another study in the same phenotype, using SCFA mixtures (rich in acetate), reported positive associations of fasting acetate with fasting fat oxidation (*r* = 0.328 *p* = 0.0228) and with resting energy expenditure (*r* = 0.349 *p* = 0.0149) [120]. In contrast, a study in healthy and T2DM subjects, acetate intravenous infusions (2.5 mmol per minute for 1 h) did not increase energy expenditure, which was partly explained by the fact that acetate might replace long chain fatty acids as preferred oxidation fuel [121]. Collectively, these studies suggested an acetate-mediated beneficial role in substrate utilization and energy expenditure in humans. However, results are inconclusive and further research is needed. In addition, the beneficial effects after distal colonic (not proximal) acetate infusions [39], together with the higher acetate release in distal colon [64] strengthened the notion that targeting the distal colonic site might increase the metabolic health effects.

#### 3.3.2. Vinegar Administrations in Humans

Studies related to vinegar effects on body weight and energy expenditure in humans are limited. Nevertheless, a few studies have reported effects on body weight. For instance, a study in individuals with obesity, a 12-week vinegar intervention significantly lowered body weight with low (0.75 g) and high (1.5 g) acetate doses versus placebo (0 g) in a dose-dependent manner [122]. In addition, a crossover study in overweight-obese subjects, the consumption of Kimchi (fermented Korean dish, unclear % of acetic acid) vs. unfermented dish reduced body fat (~1%), body weight (~1.5 kg), and BMI (0.6 kg/m^2^) after two weeks [123]. In a similar crossover study, in overweight women, fermented Kimchi (unclear % of acetic acid) decreased *Firmicutes/Bacteroidetes* ratio [124], which has been associated with weight loss [125]. In HFD-fed mice, supplementation of synthetic acetic acid (4%) and high dose of Nipa vinegar (unclear percentage of acetic acid) reduced lipid deposition, inflammation, and improved serum lipid profiles in comparison to the control. Both vinegars decreased the *Firmicutes/Bacteroid*etes ratio and increased the relative abundance of various bacterial genus including *A. muciniphila* and *Lactobacillus* among other potential acetate producers [126]. With respect to differences in acetate infusions (sodium acetate) versus vinegar (acetic acid) administrations, both the route and absorption may differ. Saunders et al. [127] reported that oral acetic acid administration was more rapidly absorbed in the stomach when compared with sodium acetate administrations, possibly through a pH-dependent mechanism, since acetic acid (unionized acetate) absorption increased when gastric pH decreased.

In previous human and rodent studies, the effects were attributed to acetic acid, either its content (%) or the presence of other bioactive components is unclear. In addition, fermented dishes (e.g., Kimchi) may have other volatile and non-volatile compounds and overall composition can vary depending on fermentation time and storage room temperature [128]. In general, acetate may modulate body weight control through different mechanisms that can affect central appetite regulation, gut-satiety hormones, and improvements in lipid metabolism and energy expenditure. However, human evidence is accumulating showing that acetate may prevent body weight gain and adiposity through increments in energy expenditure, as observed in the following acute colonic distal infusion studies. Importantly, there is a lack of longer-term human studies that investigate acetate effects on energy expenditure as a primary outcome. In the next section, we discuss acetate effects in the peripheral tissues that may collectively improve insulin sensitivity (See Table 3).

## 4. Acetate and the Inter-Organ Crosstalk and Insulin Sensitivity in Peripheral Tissues

Adipose tissue is the main organ for triacylglycerol storage in the human body and an active endocrine regulator of energy homeostasis. Therefore, metabolic derangements in adipose tissue function contribute to pathophysiology and dysregulation of glucose homeostasis and whole-body insulin sensitivity [129].

### 4.1. Acetate and Vinegar Studies and Insulin Sensitivity

Human acute acetate infusions have shown inhibitory roles in whole-body lipolysis, increase in gut-hormone release, and increase in fat oxidation and energy expenditure among other effects (See Table 2). Collectively, these effects may improve adipose tissue lipid buffering capacity, satiety regulation, oxidative capacity, and, in turn, improve whole-body insulin sensitivity and peripheral tissue functioning. In addition, vinegar administrations have reported improvements in glucose homeostasis and insulinemic profiles. Moreover, we discuss vinegar effects on glucose homeostasis and insulinemic profiles with potential T2DM treatment applications [46].

As mentioned above, oral vinegar (4%–8% acetic acid) administrations may rapidly increase circulating acetate and its co-ingestion with carbohydrates (50–75 g), which seems more effective for glucose lowering and insulinemic responses. In contrast to colonic sodium acetate infusions, oral vinegar administrations have shown improvements in glucose homeostasis and insulin profiles in healthy subjects [46,130,131]. For instance, supplementation of acetic acid (unspecified vinegar) in healthy subjects together with a test meal resulted in reduced postprandial glucose concentration (~35%, during 30–70 min), putatively, through a delayed gastric emptying [130]. Similarly, white vinegar (6% acetic acid) administrations (18, 23, and 28 mmol/L) in combination with white wheat bread (50 g) in healthy subjects lowered glycemic (highest dose at 30–45 min) and insulinemic (highest dose at 15–30 min) postprandial responses [132]. In addition, acetic acid lowered the glycemic index (GI) and increased the satiety score postprandially at 30, 90, and 120 min using a subjective rating scale [132]. Another study in healthy subjects reported that a vinaigrette (28 g white vinegar, 6% acetic acid) on a potato meal reduced GI and insulinemic index (43 and 31%, respectively) [133].

Furthermore, vinegar administration studies in individuals with metabolic alterations (e.g., impaired glucose tolerance IGT, T2DM) have been performed [46]. For instance, a study in individuals with IGT showed that wine vinegar (6% acetic acid) administration decreased arterial plasma insulin (by 33%) and increased muscle glucose uptake (by 35%) after a meal test, as compared to the placebo (50 mL water) [134]. Additionally, in T2DM individuals, oral wine vinegar administration (1.2 g acetic acid) decreased incrementalAUC 120 min of glucose (41%) only after a high GI meal test (mashed potatoes and low-fat milk) but not after a low GI meal test [135]. However, these reported beneficial effects of vinegar on glucose homeostasis in metabolically compromised individuals have not been confirmed in all studies. A study in T2DM individuals using white vinegar (1 g acetic acid) did not show any effect on postprandial glucose levels after an oral glucose load (75 g) [136]. In summary, acetate infusions and vinegar administrations have reported beneficial effects on glucose homeostasis and potentially on insulin sensitivity. However, inconsistencies exist and differences between phenotypes require further research.

### 4.2. Adipose Tissue Metabolism

#### 4.2.1. Lipolysis

Acetate administration has been shown to affect whole-body as well as intracellular lipolysis in adipocytes in in vitro and in vivo animal and human studies. Ge et al. [137] reported that acetate-mediated activation of GPR43 in 3T3-L1 adipocytes was accompanied by an inhibition of the lipolytic response within a physiological range of 100–300 µmol/L [137]. Moreover, a supraphysiological concentration of sodium acetate (4 mmol/L) induced an antilipolytic effect in murine 3T3-L1 adipocytes, via a decrease in phosphorylation of the cytosolic lipase HSL (hormone sensitive lipase) at serine residue 563 (equivalent to Ser 552 in humans) [138]. Similarly, Heimann et al. [139] described an antilipolytic effect of acetate, possibly mediated by a decrease in phosphorylation of HSL (at Ser 563) in rat and human primary (at Ser 552) adipocytes with a supraphysiological (10 mmol/L) concentration of acetate [139]. Recently, an in vitro study using differentiated human multipotent adipose-derived stem cells (hMADS) showed that acetate (1 µmol/L–1 mmol/L) decreased basal and isoprenaline-stimulated lipolysis by attenuating HSL Ser 650 phosphorylation (equivalent to Ser 660 in rats) in a GPR-dependent manner [140].

In mice, an antilipolytic effect of acetate (30% reduction of plasma free fatty acids) was observed after intraperitoneal infusions of sodium acetate (500 mg/kg), which coincided with a rise in plasma acetate after 15 min of infusion (circulating levels reached a range of 0.2–1.0 mmol/L) [137]. In HFD-fed mice, using a nanoparticle delivery system, acetate decreased lipolysis and circulating free fatty acid (FFA) after intraperitoneal injection [141]. In addition, adipose tissue mRNA expression of adipose triglyceride lipase (ATGL) was reduced.

In the human in vivo situation, a handful of studies demonstrated effects on lipolysis on the whole-body level. First, Crouse et al. [142] showed that orally administered sodium acetate (given in two doses 143 mg/kg initially and 71 mg/kg 30 min later) increased plasma acetate by three-fold to four-fold and decreased FFA plasma concentrations by 25% in healthy humans within 20 min after ingestion. Second, in healthy young subjects, an intragastric infusion of sodium acetate (12 mmol/L) significantly decreased the total AUC of circulating FFA in comparison to saline infusions during five hours after infusion [119]. Third, rectal infusion of high dose sodium acetate (180 mmol/L) decreased serum FFA in comparison to saline infusions in healthy subjects for 2 h after infusion [118]. Fourth, one-hour intravenous infusion of sodium acetate in healthy individuals increased plasma acetate from 0.19 to 0.99 mmol/L and inhibited whole-body lipolysis, as shown by the decrease in plasma glycerol and FFA concentrations [143]. Thus, both animal and human data show an antilipolytic effect of acetate that may decrease lipid overflow to peripheral insulin-sensitive tissues (e.g., skeletal muscle), which may possibly improve insulin sensitivity and decrease hypothalamic inflammation.

#### 4.2.2. Adipogenesis and Browning of Adipose Tissue

Acetate may affect the proliferation and differentiation of adipocytes, which contributes to adipose tissue morphology, browning, and function. This can induce high thermogenic activity with the potential to enhance oxidative capacity [141]. Hereby, we discuss adipogenic/browning effects of acetate mainly derived from in vitro and animal data. First, a seven-day acetate incubation (0.1 µmol/L) of 3T3-L1 pre-adipocytes increased mRNA levels of GPR43 and peroxisome proliferator-activated receptor gamma (*PPARγ*), which is a master regulator of adipogenesis. This suggests a modulatory effect in adipocyte differentiation [144,145]. In support, incubation of immortalized mice brown adipose tissue cells with a supraphysiological concentration of acetate (10 mmol/L) enhanced mRNA expression levels of *PPARγ*, and the browning markers peroxisome proliferator-activated receptor-gamma coactivator-1 alpha (PGC-1α) and uncoupling protein 1 (*UCP*1) [146]. In another in vitro study of 3T3-L1 pre-adipocytes, acetate (1 mmol/L) modulated the gene expression profile with an increase in the mRNA levels of *UCP*1, *PPAR*α, and *PPARγ*. The PR domain contains 16 proteins (PRDM16) and a cell death-inducing DNA fragmentation factor-a-like effector (CIDEA) as well as markers of beige adipocytes including transmembrane protein 26 (TMEM26) and a T-box protein 1 (TBX1) [147]. Importantly, in vitro effects were replicated in KK-Ay mice after a 16-week oral administration of acetate (0.12 g/day). However, this occurs only in epididymal adipose tissue and not in inguinal or brown adipose tissues [147].

In line, several animal studies reported comparable effects. For instance, a 16-week oral acetate supplementation (5% *weight*/*weight* diet) in HFD-fed mice increased the expressions of several key genes in epididymal adipose tissue possibly mediated via increased GPR41/GPR43 expression. The genes were involved in mitochondrial biogenesis (PGC-1α, nuclear respiratory factor 1, mitochondrial transcription factor a, beta-F1-ATPase, nuclear-encoded subunit IV, and cytochrome complex), which were all reduced by the HFD alone [87]. In HFD-fed mice, acetate (injected intraperitoneally in a nanoparticle) increased PRDM16 mRNA expression in white adipose tissue (WAT) and increased thermogenesis [141]. Recently, intermittent fasting treated mice (every other day fasting, EODF) significantly induced the expression of browning markers (*UCP1)* in subcutaneous inguinal WAT, which was proposed to occur through gut-derived acetate since browning depended on gut microbiota depletion and transplantation [61]. In support, both colonic and serum acetate levels were significantly increased after both short-term and long-term intermittent fasting (3 and 15 cycles of 24 h, respectively), which suggests an acetate-mediated browning effect [61]. Although human data are scarce, a recent cross-sectional study in morbidly obese individuals reported that elevated circulating acetate levels were positively correlated with mRNA expression of the browning marker PRDM16 in abdominal subcutaneous adipose tissue, and improved insulin sensitivity (2-h euglycemic hyperinsulinemic clamp) and changes in gut microbiota composition (e.g., increased *Firmicutes* abundance) [148].

In summary, although the human evidence is limited, acetate seems to play a role in adipogenesis and browning of WAT. Together with the antilipolytic effects, acetate may restructure adipose tissue morphology and improve adipose tissue functioning as well as energy metabolism and overall metabolic health.

#### 4.2.3. Adipose Tissue Inflammation

Adipose tissue has been recognized as an important modulator of local and systemic low-grade inflammation, which is often associated with the pathophysiology of obesity and development of insulin resistance [149]. Therefore, metabolic cues from the adipose tissue can increase the recruitment, infiltration, and activation of immune cells that can promote a proinflammatory secretory profile (low-grade inflammation) [150]. A pro-inflammatory secretory profile is partly characterized by increased pro inflammatory macrophages (M1), less anti-inflammatory macrophages (M2), and T-cell polarization [151]. Therefore, we discuss acetate mediated effects on gut-derived metabolic endotoxemia and local adipose tissue inflammation.

Under different pathological conditions, a leaky gut may occur and result in metabolic endotoxemia, characterized by high circulating lipopolysaccharide (LPS) levels, which can potentially lead to chronic low-grade inflammation. This is often observed in obesity and insulin resistance [152]. Gut-derived acetate may affect gut health via an improvement in intestinal barrier function through cross-feeding mechanisms (e.g., increased butyrate concentration) [153]. In an in vitro study, acetate (30 mmol/L) decreased LPS-stimulated secretion of the tumor necrosis factor (TNF-α) from human neutrophils by ~33% (*p* < 0.01) [154].

Furthermore, microbially-derived acetate from specific bifidobacteria protected mice against a lethal injection of Escherichia coli O157:H7 that can potentially increase LPS [155]. In a three-week oral treatment in mice, acetate added to drinking water (150 mmol/L), which increased the number and function of colonic anti-inflammatory Treg cells in a GPR43 dependent manner [156], which suggests a protective and anti-inflammatory role of colonic acetate.

Additionally, in vitro data show that acetate has the potential to decrease immune cell infiltration in the adipose tissue. First, incubation of T cells from mice with supraphysiological concentration of sodium acetate (5–20 mmol/L) promoted T-cell differentiation toward an anti-inflammatory T cell polarization (both Th1 and Th17), capable to produce anti-inflammatory cytokines such as IL-17, interferon γ, and interleukin 10 (IL-10) [157]. Authors indicated that acetate can bypass the cell surface and regulate cells that have low expression of GPR41/GPR43 [157]. These effects were putatively regulated by the activation of the mechanistic target of rapamycin (mTOR)-ribosomal S6 kinase (S6K) pathway independently of GPR41/GPR43, since both receptors are not expressed in T cells [68]. In other relevant immune cells, acetate (10 mmol/L) induced TNF-α expression/secretion in M2 but not in M1 macrophages, which showed that acetate may have a different role through the GPR43 receptor [158]. Acetate may also affect CD8 T cells, which play a regulatory role in the initiation of adipose tissue inflammation, through macrophage differentiation, activation, and migration [159]. In mice, abundance of systemic acetate in response to bacterial infections was linked to an optimal function of memory CD8 T cells through an enhanced glycolytic rate and acetylation of glyceraldehyde 3-phosphate dehydrogenase (GAPDH) [160]. In summary, acetate may have a potential role to counteract a leaky gut by preserving gut integrity and health and by immunomodulating pro-inflammatory mechanisms.

Furthermore, these putative acetate mediated effects on immune cells (M2 macrophages and T cells) may improve adipose tissue remodeling/functioning and, consequently, its function toward homeostasis.

### 4.3. Acetate and Skeletal Muscle Metabolism

Obesity is characterized by ectopic fat deposition and an altered skeletal muscle glucose and lipid metabolism, which may exacerbate insulin resistance and lead to the development of T2DM [161]. In this section, we discuss the role of acetate on muscle lipid and glucose metabolism in the context of obesity-associated derangements originated by excessive lipid overflow, a systemic low-grade inflammation, and ectopic deposition that may increase insulin resistance in the skeletal muscle.

As mentioned above, GPR41/43 are expressed in the human skeletal muscle tissue [27]. Moreover, acetate uptake has been reported in skeletal muscle tissue in rodents [34] and humans [11]. In animals, acetate uptake increased together with increments in the adenosine monophosphate (AMP)/ adenosine triphosphate (ATP) ratio (~2 min) in muscle tissue after oral injection of 10.5 mg/kg of body weight (BW). However, the absorption machinery remains unclear [34]. Intracellularly, acetate may be rapidly assimilated and metabolized through the TCA cycle in the mitochondrial matrix [162], contribute to the Acetyl-coA pool [163], and/or modulate signaling mechanisms involved in muscle lipid oxidation. For instance, an acetate intragastric infusion (5 mL/kg BW) versus water infusion for six months in obese rats, revealed an increase in AMPK activity [34]. The putative mechanism possibly occurs through the catalytic activity of acetyl-coA synthase that produces acetyl-coA and increases AMP in the cytosol. Subsequently, this increases the AMP/ATP ratio, which results in an increment of AMPK phosphorylation. In addition, myoglobin and glucose transporter 4 (GLUT4) and lipolytic gene expression (long-chain acyl-CoA dehydrogenase (LCACD), 3-ketoacyl-CoA thiolase (3KACT), and PPAR) were upregulated in both the abdominal and the foreleg muscle tissue [34].

However, no actual substrate utilization was measured. Oxygen consumption rate measurements (using metabolic cage) reported 7% higher rates in acetate-treated rats, which indicates a possible increase in the whole-body metabolic rate following intragastric acetate infusion. Furthermore, in an in vitro study, using rat skeletal muscle (L6) cells, acetic acid increased the AMP/ATP ratio and the phosphorylation of AMPK in a dose-dependent (0.05–0.5 mmol/L) and time-dependent (0–30 min) manner using physiological concentrations [33]. With respect to glucose metabolism, acetate supplementation (0.2 g acetic acid/100 g diet for 2 h) increased glycogen storage (1.1 fold) and decreased glycolysis in the gastrocnemius as compared to the control (no acetate) in Sprague-Dawley rats [164]. In support, as mentioned above, increments in the protein expression of GLUT4 [34] may suggest a modulatory role in glucose homeostasis. In summary, acetate may modulate skeletal muscle lipid and glucose metabolism possibly through activation (phosphorylation) of AMPK. Whether this affects endogenous intramuscular triglycerides (IMTG) and/or exogenous (dietary) lipid oxidation and glucose homeostasis in human muscle remains unclear.

### 4.4. Acetate and Liver Metabolism

Hepatic steatosis, if untreated, may progress toward non-alcoholic steatohepatitis (NASH) and aggravate pathophysiology in obesity and comorbidities. Therefore, adequate treatment to tackle hepatic fat accumulation is of the utmost importance. As mentioned above, the liver plays a central role in acetate metabolism, since important endogenous production occur here. Furthermore, acetate may be rapidly metabolized and used as a carbon donor for intracellular pathways including cholesterol biosynthesis, acetylation processes [165], and hepatic palmitate formation [92]. Importantly, the liver is the first organ in direct contact with microbially-produced acetate coming from the ileum and proximal colon.

From a mechanistic perspective, both in vitro and animal studies have shed light on the role of acetate in liver substrate metabolism. In Fao cells (rat hepatoma cell line), 1-h incubation with physiological concentrations of sodium acetate (100–200 μmol/l) increased AMPK phosphorylation (pThr 172) by 40% compared to control treated cells [35]. Similarly, after 2-h of oral acetate administration (16.7 mmol/L at 10 mL/kg body weight) in ICR (albino strain) mice, pAMPK and the pAMPK/AMPK ratio increased [35]. In the same study, a basic chow diet with acetic acid added to a final concentration of 0.3% for eight weeks in KK-A (y) mice showed a hypoglycemic effect, which lowers TAG and increases glycogen content in the liver, while AMPK phosphorylation was not significantly increased [35]. In HFD-fed mice, supplementation with low (0.3%) and high (1.5%) acetic acid for six weeks decreased hepatic lipid accumulation, liver lipids, and increments in expression of hepatic genes associated with fatty acid oxidation (*UCP*2, *PPAR*α, CPT1, and ACOX) [88].

Furthermore, in HepG2 cells, these effects were ablated when cells were depleted of AMPK using siRNA, which suggests an AMPK mediated mechanism [88]. In line with a modulatory role of acetate, intraperitoneal injections in rats (dose of 20 mmol/kg body weight) showed an increase in the AMP/ATP ratio in liver extracts after 15 min [166]. Additionally, in HFD-fed mice, intraperitoneal injection of acetate (nanoparticle delivery method) decreased hepatic lipid accumulation, improved hepatic function, and increased mitochondrial efficiency [141]. Acetate supplementations may also increase hepatic glycogen synthesis in the muscle, as reported in rats [167,168]. The acetate: propionate ratio may be of importance for hepatic lipid biosynthesis since propionate may favor odd chain fatty acids while acetate may favor palmitate formation [169]. Moreover, odd chain fatty acids have been linked to improvements in insulin sensitivity [170]. Moreover, in men, it has been suggested that propionate may reduce acetate utilization for liver lipid biosynthesis (fatty acid and cholesterol) [171]. However, acetate has shown antilipolytic effects at the whole-body and adipose tissue level and increases in whole-body fat oxidation (See Adipose tissue metabolism). For instance, a 3-h intragastric infusion of acetate (equivalent to the fermentation of 30 g of dietary fibers) in healthy subjects decreased plasma FFA, which possibly improved lipid profiles [119]. Importantly, SCFA reach the liver in different ratios. Therefore, single SCFA may not reflect physiological effects in the lipid profile. In general, in vitro and animal studies have provided mechanistic insight into the role of acetate in the liver, where it may increase the AMP/ATP ratio, and subsequently increase AMPK phosphorylation/activity and, thereby, affect hepatic lipid (FA oxidation) and glucose (glycogen) metabolism.

### 4.5. Acetate and Insulin Secretion in Beta Cells

Glucose homeostasis is intricately regulated via insulin, which is secreted from the beta cells in the pancreas [172]. Therefore, there is no question that modulation of GSIS has an impact on glucose homeostasis [173]. From a mechanistic perspective, murine and human beta cells express GPR (GPR41 and GPR43) [29], via which acetate has reported to regulate insulin secretion [28]. In support of this G protein-mediated signaling, GPR43-deficient mice show glucose intolerance, and reduced beta cell mass and function [174]. Additionally, a study reported that the GPR43 signaling pathway may be mediated by divergent G protein pathways that can selectively potentiate (Gα_q/11_ signaling can lead to Ca^2±^ mobilization and enhance GSIS) or inhibit (Gα_i/o_ signaling can lead to cAMP inhibition and diminish GSIS) insulin secretion in rodents [29]. In addition, acetate at physiological concentrations (70–170 μmol/l) [175] only increased in vitro insulin secretion in a murine beta cell line but not in human islets and suggested species-specific differences [29].

However, the model to induce insulin exocytosis via Ca^2±^ mobilization inside the beta cells has been a consensus for a long time. Other acetate mediated signals may indirectly contribute to insulin secretion, such as via gut-derived GLP-1 and vagal activation of the parasympathetic nervous system [176]. GLP-1 may exert a direct effect on beta cells by closing K^±^_ATP_ channel in a glucose-dependent mechanism to stimulate insulin exocytosis [177,178,179]. However, whether acetate enhances gut satiety hormones remains elusive, as discussed above. Furthermore, vagal activation increased GSIS in a rat study (See Acetate and central effects on appetite regulation) associated with an increased acetate turnover [40]. However, inconsistent results have been reported, since an in vitro study using isolated rat pancreatic islets showed that acetate (1mmol/L) strongly decreased GSIS [180]. Acetate may potentially modulate circulating levels of insulin. However, whether acetate directly affects insulin secretion via G protein-mediated signaling, or indirectly via vagal/parasympathetic activation or gut-derived hormones remains to be elucidated. With respect to alpha cells, only very few studies have investigated this, with no clear effects of acetate on glucagon release [180,181].

Overall, both human acetate colonic infusions and vinegar administrations have reported effects to improve insulin sensitivity and glucose homeostasis. From a mechanistic perspective, acetate may modulate improvements in adipose tissue functioning, as well as through an increase in oxidative capacity (e.g., muscle, liver) and modulation of GSIS in the pancreas. Collectively, these tissue-specific effects may synergistically decrease lipid ectopic deposition and contribute to body weight control and glucose homeostasis. In the next sections, we discuss the prebiotic and probiotic human administrations with potential to increase acetate production.

## 5. Prebiotic and Probiotic Administrations, Body Weight Control, and Insulin Sensitivity

In this section, we discuss prebiotic and probiotic administrations to increase microbial-derived acetate and its effects in metabolic health. First, we discuss the effect of acetogenic fiber supplementation in humans. Second, we discuss the potential of probiotic interventions to increase microbial-derived acetate production in the gut and its effects on human metabolic health.

### 5.1. Acetogenic Fibers in Human Studies

As described above, acetogenic fibers in the diet may be fermented in the colon by the microbial community and, thereby, increase colonic acetate production [182,183,184]. In this case, we show that acetogenic fibers may increase circulating acetate levels and discuss a few studies that have been linked to improvements in whole-body and peripheral insulin sensitivity (See Table 4). For instance, in healthy individuals, a drink containing lactulose (0–20 g) showed a dose effect to increase acetate concentrations with the highest dose reaching (>200 µmol/L) in plasma after 2.5 h and even 6 h after ingestion (>100 µmol/L). However, no metabolic outcome was measured [51]. In line, other acute lactulose studies in healthy individuals reported an increase in whole-body acetate turnover [185,186]. In addition, young healthy individuals consuming bread overnight (evening of previous day) supplemented with 18.4 g of arabinoxylan oligosaccharides reported increased plasma acetate levels (245 µmol/L) after 10.5 h of fasting and 2 h after a standardized breakfast (215 µmol/L) [187]. This was accompanied by improved glucose tolerance and insulin sensitivity possibly via increased gut fermentation (as shown by increased circulating SCFA and breath hydrogen levels) [187].

In overweight individuals, single oral administration of 24 g inulin in overnight-fasted overweight individuals increased serum acetate and possibly reduced ghrelin in comparison to glucose and RS as a control [109]. A study in overweight subjects, using a single administration of lactulose (30 g) significantly increased plasma acetate (333 µmol/L) in comparison to saline administration (197 µmol/L) 6 h after ingestion. Moreover, acetate turnover correlated negatively with glycerol levels (*r* = −0.78, *p* < 0.02), and a decrease in free fatty acids (35%) was observed 2 h after lactulose ingestion [188]. In hypercholesterolemic men, oat bran consumption for three weeks showed a total cholesterol lowering effect (12.8%) putatively through a greater serum acetate production as compared to wheat bran [189].

Recently, a high fiber diet (including traditional Chinese medicinal foods and prebiotics) for 12 weeks in T2DM subjects showed higher improvement in glycemic control (HbA1c < 7%) than the control diet (89% vs. 50%, respectively) [190]. Of note, in this study, an active SCFA producer (ASP) index, based on the abundance and diversity of the 15 high-fiber promoted microbial SCFA producers, was increased much more in the high fiber diet as compared to the control diet [190]. Another relevant human study showed that a intervention (30 g RS/d) improved insulin sensitivity (measured with euglycemic-hyperinsulinemic clamp), lowered circulating lipids (non-esterified fatty acids and glycerol), increased fasting ghrelin, and improved insulin sensitivity during a meal tolerance test, which was accompanied by higher AUC for acetate [11]. Despite the beneficial effects of RS, the increments in ghrelin seemed counterintuitive considering the expected higher satiety after RS. Nevertheless, some studies have linked ghrelin with increased insulin sensitivity [191,192]. Although Robertson et al. [11] found beneficial effects on insulin sensitivity in healthy individuals using the gold standard measurement, the studied population is relatively small (*n* = 10) and other RCT with acetogenic fibers within a larger population did not find beneficial effects, as further discussed below [193,194]. Moreover, a study conducted by Zhao et al. [190] differed in duration compared to Robertson et al. and, according to Zhao et al. results, T2DM subjects may need an integrative intervention, since they reported beneficial effects in the group treated with a fiber-rich diet combined with lifestyle intervention and medication (acarbose).

Although the previous studies indicate that acetate may increase in response to acetogenic fiber supplementation and improve certain metabolites, other studies reported no effects of acetogenic fibers on the metabolic profile. For instance, a 12-week galacto-ligosaccharides supplementation (15 g/day) to the regular diet in obese prediabetic individuals showed an increase in fecal *Bifidobacterium*, but no effects on acetate levels (fecal and plasma), insulin sensitivity, and energy metabolism [193]. Similarly, another 12-week galacto-ligosaccharides supplementation (5.5 g/day) in overweight/prediabetic adults improved gut microbial community (increased *bifidobacteria* and lowered *bacteroides*) and reduced inflammatory markers (including C-reactive protein and fecal calprotectin). However, acetate was not measured [195]. Taken together, the discrepancies of effects between studies may suggest the importance of the type and dose of dietary fiber as well as the metabolic phenotype studied. In the next section, we discuss the potential of probiotics to improve body weight and insulin sensitivity in humans.

### 5.2. Probiotics Body Weight Control and Insulin Sensitivity

Targeting the gut microbiota with probiotics has gained interest as a therapeutic approach to combat obesity and its comorbidities. In rodents at the genus level, *Bifidobacterium* and *Lactobacillus* have been associated with reduced weight gain and markers of adiposity [196]. In this study, we discuss both animal and human studies on probiotic interventions/associations with putatively acetate-mediated metabolic health effects.

First, a probiotic intervention in mice, (*Bifidobacterium animalis* ssp. *lactis GCL2505*) modified microbial community (increased *Bifidobacterium* and *Lactobacillus)*, increased acetate (caecal and plasma) levels and improved glucose tolerance, which was accompanied by a reduction of adipocyte cell size [13]. Second, mice colonized with *Bifidobacterium* strains (*Bifidobacterium longum* JCM 1217, *infantis* 157F, or *longum* NCC 2705) were protected against a lethal infection of Escherichia coli O157:H7 through an augmented acetate production and *Bifidobacterium* that promoted gut barrier integrity and reduced epithelial cell death [197]. Furthermore, Wrsozek et al. [198] proposed that two species, including *Bacteroides Thetaiotaomicron* (acetate producer) and *Faecalibacterium prausnitzii* (acetate consumer), might aid in maintaining epithelial homeostasis and gut health in rats.

Another important bacterium, *A. muciniphila,* has been associated with improvements in adiposity in both rodents and humans [196,199] and loss of *A. muciniphila* has been reported to impair gut integrity and increase insulin resistance in rodents [200]. *A. muciniphila* has been reported to correlate with serum acetate levels in humans, which suggests a putative role of *A. muciniphila* in acetate production and metabolic health [63]. Recently, an RCT reported that pasteurized *A. muciniphila* supplementation (3 months) in overweight/obese insulin resistant individuals improved insulin sensitivity (Homeostatic model assessment for insulin resistance (HOMA-IR), ±28.62 ±7.02%, *p* = 0.002), decreased body weight (−2.27 ± 0.92 kg, *p* = 0.091), and fat mass (−1.37 ± 0.82, *p* = 0.092) among other relevant blood markers [201]. Similarly, a 3-month supplementation (*L. casei Shirota*) improved insulin sensitivity index in metabolic syndrome individuals compared to baseline, but not different with the control [202]. Although these studies showed that the interventions were safe and provided benefits on metabolic health, a in-depth study of the microbial-derived metabolites is needed to identify the metabolic regulators. In addition, as Anhe et al. [203] suggested, the identification of these metabolites may provide a safer therapeutic option and overcome the limitations of probiotic supplementations. In addition, a metanalysis of randomized controlled trials reported that probiotic supplementation (e.g., Lactobacillus reuteri, Lactobacillus gasseri) can significantly reduce body weight ((95% CI) −0.60 kg), BMI ((95% CI) −0.27 kg/m^2^), and fat percentage ((95% CI) −0.60%) in overweight/obese individuals. However, the effect sizes were small [204]. *L. plantarum* reduced glucose and homocysteine levels significantly after 12 weeks in premenopausal insulin resistant women [205]. In addition, a combination of probiotics with prebiotics may provide a synergistic effect. For instance, a symbiotic administration (*Lactobacillus* and fructo-ligosaccharides) in insulin-resistant individuals showed improvements in fasting levels of glucose and HOMA-IR in comparison to the placebo after a 28-week treatment [206].

Future probiotic studies should identify the mechanisms and factors that ensure its efficacy as well as which symbiotic mixtures may have a better synergistic effect [207,208]. Although these studies report benefits on insulin sensitivity (HOMA-IR) and glucose levels with potential to improve metabolic health following probiotic interventions, it remains unknown whether these effects are acetate-mediated. Nevertheless, effects were mediated by specific species such as *Bacteroides Thetaiotaomicron* and *A. muciniphila*, which are important acetate producers.

Exogenous acetate production includes vinegar as well as the supplementation of acetogenic fiber and probiotics. Acetogenic fiber characteristics (e.g., length, glycosidic bond configuration) may determine acetate production. Endogenous acetate production occurs in all tissues but predominantly in the liver. Microbial acetate is mainly produced in the colon. Colonic absorption and acetate systemic concentrations may differ between colonic production sites (proximal/distal). Furthermore, acetate may increase GLP-1 and PYY secretion in the colon. Systemic acetate may improve metabolic health through improvements in adipose tissue functioning (antilipolytic/anti-inflammatory effects), insulin sensitivity, and oxidative capacity (e.g., liver, skeletal muscle) increments in satiety (central nervous system) as well as modulation of insulin secretion (pancreas). Solid lines indicate well-studied effects of acetate. The dashed lines indicate more inconsistent findings.

## 6. Conclusions and Perspectives

**Figure 1:**
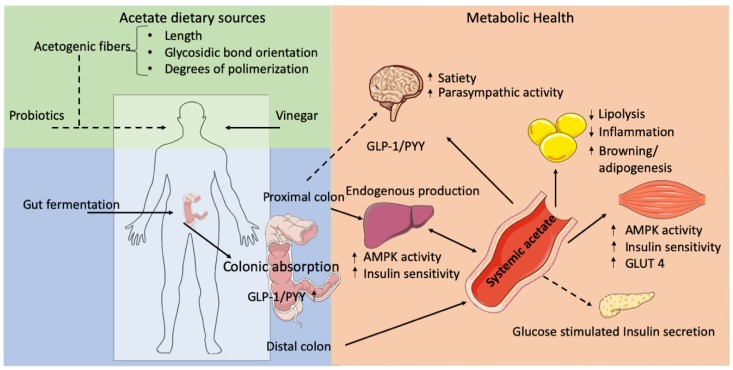
Acetate sources and acetate-mediated effects in metabolic health. Exogenous acetate production includes vinegar as well as the supplementation of acetogenic fiber and probiotics. Acetogenic fiber characteristics (e.g. length, glycosidic bond configuration) may determine acetate production. Endogenous acetate production occurs in all tissues but predominantly in the liver. Microbial acetate is mainly produced in the colon. Colonic absorption and acetate systemic concentrations may differ between colonic production sites (proximal/distal). Importantly, acetate may increase GLP-1 and PYY secretion in the colon. Systemic acetate may improve metabolic health via improvements in adipose tissue functioning (antilipolytic/anti-inflammatory effects) insulin sensitivity and oxidative capacity (e.g. liver, skeletal muscle) increments in satiety (central nervous system) and modulation of insulin secretion (pancreas). Solid lines indicate well-studied effects of acetate, dashed line indicate more inconsistent findings.

From a mechanistic perspective, a vast wealth of animal data suggests that acetate has an important regulatory role in body weight control, and insulin sensitivity through effects on lipid metabolism and glucose homeostasis (as summarized in Figure 1). Current evidence of acetate-mediated effects on metabolism emphasizes the need for well-controlled human intervention studies that ensure an efficient administration of acetate (considering location and concentration) in a physiological and appealing manner. Under this premise, prebiotic supplementation has been conducted. However, this process had inconsistent results with regard to increasing colonic/systemic acetate production. Nevertheless, a few human studies have shown the capacity to improve markers of whole-body insulin sensitivity [11,187,190]. Similarly, a few probiotic human studies, with the potential to increase acetate production, have reported improvements in whole-body insulin sensitivity [202,206]. While the effect of changes in acetate levels to the observed metabolic phenotype in these studies remains unclear, in light of the evidence presented in this report, it is reasonable to hypothesize that acetate acts as a direct mediator of these effects. With respect to oral acetate administrations (vinegar), improvements on glucose homeostasis have been reported, and attributed to acetic acid. Future vinegar supplementations should specify its composition (e.g., acetic acid percentage, polyphenols). Moreover, future prebiotic/probiotic/vinegar studies should consider that responses may differ between healthy individuals and pre-diabetic individuals, as shown in vinegar administrations.

## Figures and Tables

**Table 1:** Circulating acetate in humans.

Condition	Site and Average (SEM) Concentrations	Population	Study
Fasting	Superior mesenteric vein 50.4 ± 11.3 µmol/L Inferior mesenteric vein 102.7 ± 27.2 µmol/L Portal vein 41.4 ± 7.8 µmol/L Hepatic vein 23.6 ± 4.8 µmol/L Radial artery 21.8 ± 7.6 µmol/L	Healthy/Overweight upper abdominal surgery patients (54–75 years)	Neis et al. (2018) [64]
Fasting	Peripheral vein 44 ± 4.4 µmol/L	Healthy/Ileostomy (56–80 years) patients	Scheppach et al. (1991) [65]
Fasting	Small intestine 77.6 ± 3.23 mmol/kg Large intestine 53.72 ± 9.87 mmol/kg Portal vein 258 ± 40.13 µmol/L Hepatic vein 115 ± 28.20 µmol/L Peripheral vein 70 ± 18.55 µmol/L	Sudden death victims (16–89 years)	Cummings et al. (1987) [22]
Fasting	Peripheral vein 53.8 ± 4.44 µmol/L Peripheral artery 125.6 ± 13.4 µmol/L	Healthy patients (19–41 years)	Pomare et al. (1985) [51]

Abbreviations: SEM, standard error of the mean.

**Table 2:** Effects of sodium acetate infusions in humans at different administration sites.

Dose	Primary Outcome	Subjects	Administration Site	Effects on Lipid/Glucose Metabolism	Study
SCFA infusion mixtures rich in acetate (24 mmol/L acetate, 8 mmol/L propionate, and 8 mmol/L butyrate) and propionate (18 mmol/L acetate, 14 mmol/L propionate, and 8 mmol/L butyrate)	Fat oxidation and energy expenditure	Overweight/obese men (*n* = 12)	Colonic infusions	Attenuation of whole-body lipolysis ↑ Fat oxidative capacity Fat oxidation and energy expenditure related to increments in fasting acetate ↑ Fasting and postprandial PYY No effects on insulin and glucose	Canfora et al. 2017 [120]
180 mmol/L sodium acetate	Fat oxidation and energy expenditure	Overweight/obese men (*n* = 6)	Proximal and distal colonic	↑ Fasting fat oxidation ↑ Postprandial glucose and insulin Tendency to decrease TNF-α ↑ Fasting peptide YY	Van der Beek et al. (2016) [39]
140 mmol/L in 90-min sodium acetate	Peripheral uptake	Overweight normoglycemic and hyperglycemic subjects (*n* = 9 vs. 9)	Intravenous	No difference in acetate clearance between individuals with normal (NI) and high (HI) insulin levels. ↑ FFA rebound in NI than HI.	Fernandes et al. (2012) [113]
60 mmol/L (rectal), 20 mmol/L (intravenous) sodium acetate	Gut derived hormone secretion	Hyperinsulinaemic females (*n* = 6)	Rectally and intravenous	in PYY/GLP-1 after rectal infusions and decrease in TNF	Freeland et al. (2010) [112]
12 mmol/L per hour	Hepatic glucose production	Healthy subjects (*n* = 6)	Intragastric	↓ Circulating FFA No effect on hepatic glucose production	Laurent et al. (1995) [20]
800 mL rectal infusions with 180 mmol/L	Glucose homeostasis	Healthy subjects (*n* = 6)	Rectal infusion	No effects on insulin and glucose ↓ Circulating FFA	Wolever et al. (1989) [118]

Abbreviations: SCFA, short chain fatty acids. PYY, peptide YY. GLP-1, glucagon like-peptide 1. TNF-α, Tumor necrosis factor. FFA, free fatty acid.

**Table 3:** Vinegar administrations in humans.

Dose, Vinegar Type	Primary Outcome	Subjects	Effects	Study
Unspecified vinegar	G&IR	Healthy (22–51 years), seven females (*n* = 10)	↓ 35% postprandial glucose Delayed gastric emptying	Björck et al. 2005 [130]
White vinegar 6% acetic acid	G&IR	Healthy (19–27 years), 10 females (*n* = 12)	↓ Glucose and Insulin	Dimitriadis et al. (2015) [132]
White vinegar 6% acetic acid	G&IR	Healthy (19–32 years), 10 females (*n* = 13)	↓ Glycemic index and Insulinemic index (43% and 31%, respectively)	Diakoumoupolou et al. (2010) [133].
Wine vinegar 6% acetic acid	Muscle glucose metabolism Circulating lipids endothelial function	Individuals with impaired glucose tolerance (26–66 years), four females (*n* = 8)	↑ Muscle blood flow 33% ↑ Muscle glucose uptake 35%	Luc van Loon et al. (2012) [134]
Wine vinegar 1.2 grams acetic acid vs. placebo	G&IR	TD2M individuals (*n* = 8 vs. 8) (40–78 years), 4 females	↓ iAUC_120_ Glucose 41%	Crovetti et al. (1995) [135]
White vinegar 1-gram acetic acid	G&IR	TD2M male individuals (*n* = 12) (63–67 years)	No effect	Haldar et al. (2016) [136]

Abbreviations: T2DM, type 2 diabetes mellitus. iAUC, incremental area under the curve. G&IR, glycemic and insulinemic response.

**Table 4:** Acetogenic fiber administrations with effects on glucose homeostasis and metabolic health.

Participants	Non-Digestible Carbohydrate	Design	Effects	Study
T2DM individuals (*n* = 43)	High fiber diet (*n* = 27) Control diet (*n* = 16)	12 weeks RCT	Higher improvement in glycemic control (HbA1c < 7%) in treatment (89% vs. 50%, respectively) ↑ abundance in SCFA microbiota producers in treatment	Zhao et al. (2018) [190]
Healthy adults (*n* = 10)	20 grams resistant starch 10 grams (3 times/day)	4 weeks, placebo/controlled (20 grams digestible starch)	Improved whole-body insulin sensitivity (euglycemic-hyperinsulinaemic clamp) by 13% (*p* < 0.05)	Robertson et al. (2005) [11]
Hypercholesterolemic men (*n* = 20)	Oat bran (47.4 grams/day) wheat bran (control) (41.8 grams/day)	RCT 3 weeks	↓ Total cholesterol (12.8%) Linked to high acetate in plasma ↑ Higher acetate in treatment	Bridges et al. (1992) [189]
Healthy individuals (*n* = 14)	0, 10, and 20 grams lactulose Control (water)	Single dose	No effects in glucose homeostasis investigated Dose effect in acetate levels ↑ fermentation after 6 h	Pomare et al. (1985) [51]
Healthy individuals (*n* = 19)	Arabynoxylan oligosaccharides (AXOS) (8.9 grams) High AXOS (18.4 grams)	Randomized cross-over Overnight administration	↑ improvement in glucose tolerance ↑ improved insulin sensitivity index with High AXOS Dose-effect increase in plasma acetate (>200 µmol/L)	Boli et al. (2016) [187]
Overweight obese individuals (*n* = 53)	Pea fiber (15 grams/day) Control (no fiber)	RCT 12 weeks	No effects in glucose homeostasis ↑ Fecal acetate No effects on plasma acetate	Mayengbang et al. (2017) [194]
Lean/overweight individuals (*n* = 12, 13 respectively)	Inulin (24 grams) Control (glucose)	Cross-overSingle dose	↑ Acetate in plasma Possibly linked to ghrelin reduction	Rahat-Rozenbloom et al. (2016) [109]
Overweight individuals (*n* = 8)	Lactulose (30 grams)	Single dose	↑ Acetate in plasma Correlation of acetate and in lipolysis (glycerol turnover)	Ferchaud-Roucher et al. (2005) [188]

Abbreviations: T2DM, type 2 diabetes mellitus. RCT, randomized controlled trial. HbA1c, glycated hemoglobin. SCFA, short chain fatty acids.

## Abbreviations

GLP-1	glucagon-like peptide 1
PYY	peptide YY
AMPK	AMP-activated protein kinase
GLUT	glucose transporter
